# Symptoms awareness, emergency medical service utilization and hospital transfer delay in myocardial infarction

**DOI:** 10.1186/s12913-018-3312-6

**Published:** 2018-06-25

**Authors:** Cézar E. Mesas, Ricardo J. Rodrigues, Arthur E. Mesas, Vivian B. R. Feijó, Lucas M. C. Paraiso, Gabriela F. G. A. Bragatto, Viviane Moron, Marcos H. Bergonso, Laercio Uemura, Cintia Magalhães Carvalho Grion

**Affiliations:** 1 Centro de Ciências da Saúde, Universidade Estadual de Londrina, Rua Robert Koch 60, Vila Operária, Londrina, Paraná 86038-350 Brazil; 2 Santa Casa de Londrina, Londrina, Brazil

**Keywords:** Myocardial infarction, Reperfusion time, Mortality

## Abstract

**Background:**

The length of time between symptom onset and reperfusion therapy in patients with ST-segment elevation acute myocardial infarction (STEMI) is a key determinant of mortality. Information on this delay is scarce, particularly for developing countries. The objective of the study is to prospectively evaluate the individual components of reperfusion time (RT) in patients with STEMI treated at a University Hospital in 2012.

**Methods:**

Medical records were reviewed to determine RT, its main (patient delay time [PDT] and system delay time [SDT]) and secondary components and hospital access variables. Cognitive responses were evaluated using a semi-structured questionnaire.

**Results:**

A total of 50 patients with a mean age of 59 years (SD = 10.5) were included, 64% of whom were male. The median RT was 430 min, with an interquartile range of 315–750 min. Regarding the composition of RT in the sample, PDT corresponded to 18.9% and SDT to 81.1%. Emergency medical services were used in 23.5% of cases. Patients treated in intermediate care units showed a significant increase in SDT (*p* = 0.008). Regarding cognitive variables, PDT was approximately 40 min longer among those who answered “I didn’t think it was serious” (*p* = 0.024).

**Conclusions:**

In a Brazilian tertiary public hospital, RT was higher than that recommended by international guidelines, mainly because of long SDT, which was negatively affected by time spent in intermediate care units. Emergency Medical Services underutilization was noted. A patient’s low perception of severity increased PDT.

**Electronic supplementary material:**

The online version of this article (10.1186/s12913-018-3312-6) contains supplementary material, which is available to authorized users.

## Background

Cardiovascular diseases are the leading cause of death in industrialized countries. According to the Brazilian Unified Health System’s Database, cardiovascular disease accounted for 10% of all hospitalizations and more than one-third of deaths in Brazil [1]. Acute coronary syndromes (ACS) represent a major cause of hospitalization and are the third leading cause of hospitalization in the Unified Health System [2].

The majority of deaths from ST-segment elevation acute myocardial infarction (STEMI) occur in the first hours of disease manifestation, with 40 to 65% in the first hour and approximately 80% in the first 24 h [3]. A robust body of evidence indicates that rapid restoration of flow in coronary artery in STEMI patients reduces morbidity and mortality [4–6]. Studies show that every 30 min delay in reperfusion time reduces life expectancy by 1 year [7] and that when the flow is restored after 6 h, there is little decrease in mortality [8, 9].

Even though the interval between symptom onset and reperfusion therapy in STEMI patients is recognized as a prognostic determinant [4, 9], little is known about the effects of individual components in the delay. Reperfusion time (RT) or total ischemia time, recorded from the onset of symptoms to restoration of coronary flow, consists of two main components: Patient Delay Time (PDT - symptom onset to first medical contact) and System Delay Time (SDT - from first medical contact to arterial reperfusion) [4]. When the reasons for the delay in patients seeking help are evaluated, a multiplicity of factors is revealed, including the context in which the symptoms appear, socioeconomic variables, cognitive and emotional responses and the reactions of witnesses [10–12].

It is recognized that the treatment of STEMI patients is complex and involves characteristics unique to local communities and healthcare systems. We sought to investigate such reality, that is poorly understood, especially in low and middle-income countries.

## Methods

The aim of this study is to characterize the individual components of RT in patients with STEMI, analyzing variables influencing PDT (clinical, demographic, socioeconomic and cognitive/behavioral factors) and SDT, including pre-hospital end in-hospital (D2B) times.

### Study design and population

This short-term longitudinal descriptive study evaluated all consecutively patients with STEMI seen at the University Hospital of Londrina (Londrina State University) during the year of 2012, who underwent reperfusion therapy by primary percutaneous coronary intervention (PCI). This PCI-capable center is a medium size (313 beds), teaching hospital, and is the preferential destination for patients with STEMI, users of the Brazilian Unified Health System, for a population of over 1.000.000 inhabitants.

All patients underwent primary reperfusion therapy by PCI for STEMI, according to criteria established by current guidelines for the management of patients with ST-elevation myocardial infarction [6]. Exclusion criteria were: 1) Inability or refusal by the patient or legal representative to provide consent and/or answer the semi-structured questionnaire; 2) Age < 18 years; and 3) Prior reperfusion therapy less than 6 months previously.

### Data collection

This PCI-capable center is a public, medium size teaching hospital (313 beds, with an average of 25 beds for patients with heart disease) and is the preferential destination for patients with STEMI for a local population of over 1.000.000 inhabitants, 60% of whom are users of the Brazilian Unified Health System (SUS). Given the insufficient availability of beds, many patients are referred to smaller, local hospitals, where they are treated with PCI or thrombolysis. These factors, along with late referral, can explain a rather lower than expected volume of primary PCI (less than 70 per year).

A daily active search was conducted, and all patients who met the inclusion criteria were included on a consecutive basis. Demographic and socioeconomic data (age, gender, education and family income) were collected by means of interview and review of medical records, along with information regarding clinical conditions (presence of previous cardiovascular disease, smoking, hypertension, diabetes mellitus and dyslipidemia).

Patients answered a semi-structured questionnaire to evaluate the reasons and personal perceptions that affected the decision time to seek help after symptom onset. An adaptation of the Response to Symptoms Questionnaire was used [11]. As no appropriate questionnaire validated for the Portuguese language was available during the study design, each item from the Response to Symptoms Questionnaire was translated and used as an individual question for descriptive purposes, i.e., formal translation, transcultural adaption and validation of the RSQ to Brazilian Portuguese is still lacking. Examples of answers included: “I didn’t think it was serious” and “I was worried about troubling others so didn’t ask for help.” For more detail, the English (Additional file 1) and Portuguese version (Additional file 2) of questionnaire are available as additional files.

All study information was collected during hospitalization. Information that could not be obtained from the questionnaire or medical records was labeled as missing data.

The time-based variables recorded were as follows:

1. Reperfusion time (RT): time from symptom onset to balloon inflation in the catheterization laboratory, representing total ischemic time.
2. Decision time (DT): time from symptoms onset to the decision to seek the medical service.
3. Patient delay time (PDT): time from symptoms onset to first medical contact (FMC) via telephone or directly.
4. System delay time (SDT): time between first medical contact (phone or directly) and balloon inflation in the catheterization laboratory (CL). SDT is the sum of the pre-hospital and door-to-balloon times.
5. Pre-hospital time (PHT): time from FMC to arrival at the PCI-capable hospital (University Hospital of Londrina).
6. Door-to-door time (D2D): time between the patient’s arrival at the intermediate care unit (unit that precedes the PCI-capable hospital) and at the PCI-capable hospital.
7. Door-to-balloon time (D2B): time between patient arrival at the PCI-capable hospital and balloon inflation in the catheterization laboratory (CL).
8. Door-to-ECG time: time between patient arrival at the PCI-capable hospital and electrocardiogram (ECG) acquisition.
9. CL activation time (D2Page): time between patient arrival at the destination hospital and CL activation.
10. CL response time: time between prescription of PCI to balloon inflation in the CL.

### Statistical analysis

Time variables (in minutes) were initially analyzed for conformity to the normal distribution assumptions using the Shapiro-Wilk test, which revealed that none were normally distributed. Therefore, continuous variables were described as medians and interquartile ranges (ITQ). Comparisons of times according to the variables of interest were performed using the non-parametric Mann-Whitney test.

To analyze the compositions of pre- and in-hospital time and of RT according to their components, the percentage fraction of each component for each patient was initially calculated, and the mean percentages were obtained for the total sample based on these values.

Univariate analyses for PDT included the independent variables age, gender, income, education, cognitive responses to symptoms, responses of others to symptoms, pain severity, number of risk factors for coronary disease, and whether the symptoms were witnessed by another person.

Categorical data are expressed as frequencies and presented in tables. Categorical variables were analyzed using the chi-square test, and the results are expressed as odds ratios (OR) and 95% confidence intervals (95% CI). The significance level was set at 5%, and analyses were performed using SPSS version 20.0 (SPSS Inc., Chicago, IL).

## Results

During the study period, 50 STEMI patients were admitted on a consecutive basis with indications for reperfusion by PCI, and no patient was excluded. Table 1 shows the frequencies of the main socioeconomic and clinical variables. Of the 50 patients studied, 32 (64%) were male, and the mean age was 59 years (standard deviation SD = 10.5 years). Three out of every four patients reported having completed primary education, and half reported a monthly income of less than three times the minimum wage. A total of 74% had up to one coronary atherosclerosis risk factor, while two or more risk factors were found in 26%. Only 23.5% of patients called the emergency medical services (EMS). The median EMS response time was 30 min (ITQ: 15–99). The times recorded between symptom onset and balloon inflation (RT) are shown in Table 2. The PDT corresponded to 18.9% of RT (45 min, IQT: 30–140), while the SDT accounted for 81.1% of total time, with a median of 319 min (ITQ: 220–615).

**Table 1:** Comparison of patient delay time^a^ according to socioeconomic and clinical variables

Variable	N (%)	Median patient delay time (ITQ)	*p* value^†^
Gender			
Female	18 (36)	35 (25–70)	0.10
Male	32 (64)	70 (30–150)	
Age			
< 65 years	37 (74)	57 (30–145)	0.86
≥ 65 years	13 (26)	45 (30–105)	
Monthly income			
≤ 3 minimum wages	22 (48)	55 (27–157)	0.35
< 3 minimum wages	24 (52)	40 (30–105)	
Education			
Primary	35 (73)	45 (30–140)	0.99
Secondary	13 (27)	35 (20–120)	
Pain severity			
≤ 6	43 (91)	105 (20–525)	0.66
> 6	4 (9)	45 (30–135)	
Number of risk factors			
< 2	20 (40)	42 (30–112)	0.49
≥ 2	30 (60)	70 (30–141)	

*ITQ* interquartile range

^†^Mann-Whitney test

^a^minutes

**Table 2:** Description of reperfusion time^a^ and its components in STEMI patients treated in the University Hospital of Londrina in 2012

Variable	Minimum	25th percentile	Median	75th Percentile	Maximum
Reperfusion time	100	315	430	750	4205
Decision time	0	10	20	60	840
Patient delay time	0	30	45	140	870
System delay time	75	220	319	615	4085
Pre-hospital time	0	75	197	420	3141
Door-to-door time	0	80	179	405	1701
Door-to-balloon time	42	81	125	212	3520

^a^minutes

The univariate analyses for PDT included the independent variables age, gender, income, education, number of risk factors for coronary disease, pain severity, cognitive responses to symptoms, responses of others to symptoms, and whether the symptoms were witnessed by another person. The data are presented in Tables 1, 3 and 4.

**Table 3:** Comparison of patient delay time^a^ according to reported cognitive response

Cognitive response	N (%)	Median patient delay time (ITQ)	*p* value^†^
Told a family member			
Yes	19 (38)	45 (30–120)	0.67
No	30 (62)	50 (30–180)	
Was scared			
Yes	13 (27)	40 (20–120)	0.26
No	34 (73)	52 (30–150)	
Didn’t think it was serious			
Yes	11 (22)	150 (70–180)	0.024
No	38 (78)	40 (30–120)	
Didn’t think it lasted long			
Yes	9 (19)	40 (30–60)	0.42
No	38 (81)	57 (30–141)	
It had occurred before and always passed			
Yes	3 (6)	10 (10–45)	0.087
No	46 (94)	57 (30–141)	
Didn’t know it was important to seek help			
Yes	2 (4)	135 (120–150)	0.83
No	47 (96)	45 (30–140)	

*ITQ* interquartile range

^†^Mann-Whitney test

^a^minutes

**Table 4:** Comparison of patient delay time^a^ according to others’ responses to symptoms

Responses of others to symptoms	N (%)	Median (ITQ)	*p* value^†^
Witnessed			
Yes	36 (74)	57 (30–145)	0.13
No	13 (26)	40 (10–120)	
Suggested seeking help			
Yes	29 (59)	45 (25–120)	0.24
No	20 (41)	65 (30–145)	
Called ambulance			
Yes	10 (20)	47 (30–140)	0.93
No	39 (80)	45 (30–141)	

*ITQ* interquartile range

^†^Mann-Whitney test

^a^minutes

**Fig. 1:**
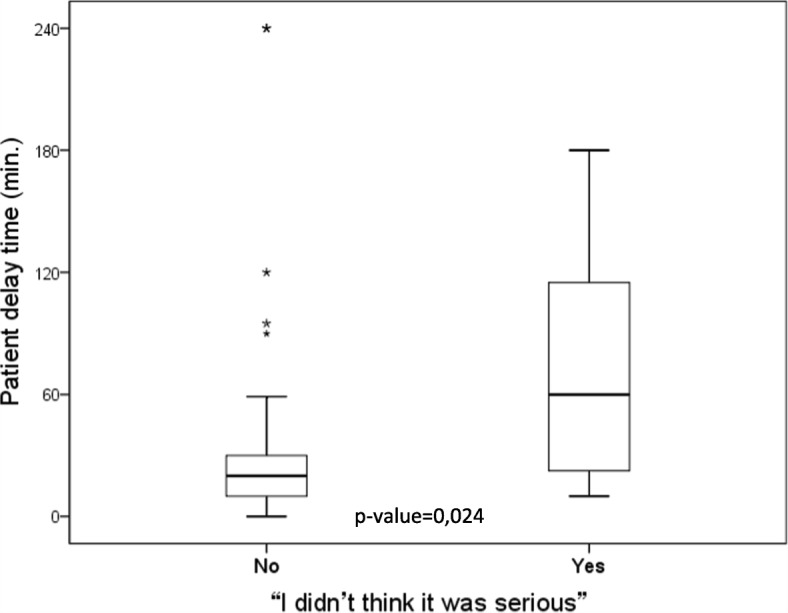
Comparison of patient delay time according to the cognitive response “I didn’t think it was serious”

Regarding cognitive variables, PDT was approximately 40 to 60 min longer among those who answered “I didn’t think it was serious” (*p* = 0.024; Table 3, Fig. 1), although this had no effect on the reperfusion time (*p* = 0.78). Dividing the PDT into two groups (< 60 min or ≥ 60 min), the cognitive response “I didn’t think it was serious” was associated with a higher risk of delay (OR = 6.00; 95% CI: 1.35 to 26.65). Regarding other cognitive responses or responses of others to symptoms, no significant differences were observed in terms of seeking help or making the first medical contact.

In cases in which the patient was taken directly to the PCI-capable hospital (University Hospital of Londrina), it was found that the SDT was made up of 54.3% PHT and 45.7% of D2B time. When the patient was treated in an intermediate care unit (basic health unit or secondary hospital), the D2D (the difference between arrival at the intermediate care unit and arrival at the destination hospital) accounted for 77% of the PHT.

**Fig. 2:**
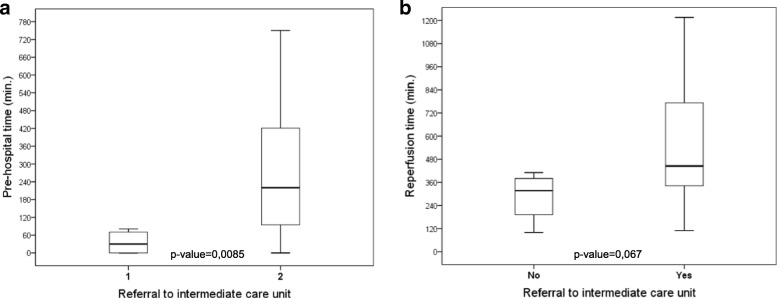
Effect of patient’s referral to an intermediate care unit prior to the PCI-capable hospital in pre-hospital time (**a**) and reperfusion time (**b**)

Table 5 contains the variables relating to the flow of patients and their relation to PHT: form of first medical contact (phone or attendance at the medical service), transport mode to the first medical service (own vehicle or ambulance) and presence of intermediate care unit between first medical contact and the PCI-capable hospital. The median PHTs were 220 min (ITQ: 90–422) for patients who were referred to an intermediate care unit and 30 min (ITQ: 0–75, *p* = 0.008) for those who spontaneously attended or were sent directly to the referral hospital (Fig. 2a). Although non-significant, this difference showed a trend to increase the RT for those sent to intermediate care units (*p* = 0.067, Fig. 2b).

**Table 5:** Comparison of pre-hospital time^a^ according to referral hospital access variables

Variable	N (%)	Median (ITQ)	*p* value^†^
Form of first medical contact			
Telephone	10 (22)	78 (39–242)	0.17
Direct	35 (78)	220 (100–422)	
Transport to first medical service			
Own vehicle	36 (76)	231 (105–421)	0.62
Ambulance	11 (24)	75 (39–406)	
Intermediate care unit			
Yes	43 (91)	220 (90–422)	0.0085
No	4 (9)	30 (0–75)	

*ITQ* interquartile range

^a^minutes

^†^Mann-Whitney test

Table 6 outlines the composition of D2B time and the distribution of delays (D2Page and CL response time) and shows that the response time of the CL represented 57.5% of D2B, with the ITQ ranging between 30 and 107 min. Table 7 presents the bivariate analyses for D2B and the independent variables: type of on-duty physician (cardiologist, non-cardiologist) and attendance during business hours versus nighttime, weekends or public holidays. There were no significant differences for these variables.

**Table 6:** Fractionation of door-to-balloon time^a^ in STEMI patients

Variable	Fraction (%) of door-to-balloon time	Median (ITQ)^a^
Door-to-page time	42.5	40 (15–95)
CL response time	57.5	70 (30–107)
Total door-to-balloon time	100.0	125 (81–212)

*ITQ* interquartile range

^a^minutes

**Table 7:** Comparison of in-hospital time according to treatment variables

Variable	Median (ITQ)^a^	*p* value^†^
Type of on-duty physician		
On-call cardiologist	112 (70–189)	0.48
On-site cardiologist	91 (88–225)	
Non-cardiologist	186 (96–224)	
Business hours		
Yes	112 (88–210)	0.52
No	160 (78–204)	
Directly sought the University Hospital		
Yes	202 (127–227)	0.37
No	123 (81–210)	

*ITQ* interquartile range

^a^minutes

^†^Mann-Whitney test

## Discussion

This study evaluated the components of RT and the variables related to it in STEMI patients treated by primary PCI in the largest public hospital in the state of Paraná, Brazil, during 2012. Confirming results from other records, the RT found in this “real world” scenario was above that recommended by national and international guidelines [13–15]. The SDT time was largely responsible for the delay in RT, particularly when the patient sought or was initially taken to an intermediate care unit.

In general, patients do not seek medical care until 1.5 to 2 h after the onset of pain. This reality has not changed significantly in the last 10 years, despite the implementation of specific public policies [16]. Sullivan et al. found a mean time from onset of symptoms to hospital arrival that ranged from 1.5 to 6 h and estimated that each 30-min delay increases mortality by infarction in 1 year by 7.5% [17]. Terkelsen et al. evaluated 6209 patients in Denmark between 2002 and 2008 and found a mean PDT of 74 min, corresponding to 43% of RT (172 min) [4].

The PDT observed in this study had a median of 45 min, with 40% of patients seeking help in the first 30 min. Although less than or comparable to previous studies, this delay is still significantly longer than the 5 min recommended by the American Heart Association [6, 15, 18]. Proportionally, while PDT accounted for only 18.9% of total RT, in the United States and Europe, this proportion is approximately 40% [4], a value possibly related to the lower SDT in these countries.

Previous studies have identified reasons for the increase in the patient’s DT, the main component of PDT: the perception that the symptom is self-limiting, attributing the symptoms to other conditions, fear of disturbing others, fear that the symptoms are a false alarm, lack of knowledge of the importance of quick action and lack of awareness that one should call the EMS [18, 19]. In this study, the only variable related to longer PDT was the low perception of severity by the patient (“I didn’t think it was serious”). Similar data were reported by Leslie et al. [18] This information reinforces the importance of public education initiatives regarding the recognition of ACS symptoms and actions to take when confronted by them.

Even though the variables female gender, black race, advanced age and low socioeconomic status contributed to an increase in PDT in other studies [3], in the present study, these variables had no effect, which underscores the importance of the individual characteristics of each community. Despite the relatively small sample size, our data show a subset of STEMI patients with educational background and income considerably lower than those observed in developed countries. This is representative of most of the Brazilian population and many other emerging economies.

Guidelines recommend that all patients with suspected ACS use the EMS [14, 15]. In the present study, this service was activated by only 23.5% of patients. This figure contrasts with the results of the American ACTION registry [20], published in 2011, in which 60% of 37,643 patients used this means of access to the health system. The reasons for the population’s low use of EMS were not analyzed, which suggests the need for future studies focusing on such issues.

In this study, only 10% of patients were taken directly to the referral service for primary PCI, whereas the others were first seen in intermediate care units (basic health units, secondary hospitals or the emergency rooms of cities in the region). The PHT of patients who went directly to the referral hospital was significantly lower than the time recorded for those who went through intermediate care units. D2D time accounted for 77% of PHT in these patients. A similar result was found by Sorensen et al., who found a mean PHT of 92 min for French patients who went directly to the referral service and a recorded time of 153 min for those who went through intermediate care units (*p* = 0.002) [21].

According to the Brazilian National Center for Cardiovascular Interventions registry for 2006 to 2010, in which 20.004 patients were studied, the mean D2B time in Brazil was 2 h [22], a value similar to that found in our study (median 125 min). In contrast to the findings of the ACTION registry [20], in our study, the patient’s mode of transport to the referral service did not affect door-to-balloon time. The time to ECG acquisition in the referral service was 11 min (ITQ: 6–30), which was very close to the recommended guidelines (10 min). However, there was a significant delay between ECG acquisition and activation of the catheterization laboratory team; this figure was not affected by the type of on-duty doctor, the time of day or day of the week of treatment. This information differs from that reported by Bradley et al., who found in a registry of 365 hospitals that SDT was significantly lower on weekdays or during business hours and when the hemodynamic team was called into action by the on-duty physician, without the mandatory involvement of a cardiologist [12]. The CL response time accounted for 57.5% of the total D2B time.

Our study was limited by the small sample size and by not including a smaller but significant number of patients treated in other public and private hospitals of the metropolitan area. However, in Brazil, and probably many other developing economies, most of the population depend on public, teaching or philanthropic hospitals, as ours [1].

Regarding the patient’s cognitive responses, we examined the answers for each question of the RSQ freely translated into Brazilian Portuguese, as formal translation and validation were not available. However, all questions were easily understood by the patients and we believe that these results might improve our understanding of patient-related components of the delay.

Recognition that SDT increases significantly when patients are directed to intermediate care units reinforces the need to improve treatment flow. The use of pre-hospital ECG (in the ambulance and in primary care units), facilitated by portable devices and telemedicine capabilities, can optimize the flow of patients for whom reperfusion is indicated, or who are at higher risk, to PCI-capable hospitals. Strategies such as these can also reduce D2B time because activation of the CL team could be anticipated in cases where primary PCI is clearly indicated.

## Conclusions

In this “real world” scenario of a medium size, Brazilian public hospital, EMS was largely underused, and RT was higher than that recommended by international guidelines, mainly because of the increase in SDT, which was negatively affected by time spent in intermediate care units. A patient’s low perception of severity might have increased PDT. Our results suggest that initiatives such as public education campaigns and patient flow optimization, encouraging the use of the EMS and pre-hospital ECG to avoid unnecessary delays in intermediate care units, may have beneficial effects on the reduction of RT in patients with STEMI.

## Additional files

Additional file 1 Response to Symptoms Questionnaire (English version). Version of symptons questionnaire modified by Dracup K and Moser DK. Beyond sociodemographics: Factors influencing the decision to seek treatment for symptoms of acute myocardial infarction. (DOCX 15 kb)

Additional file 2 Response to Symptoms Questionnaire (Portuguese version). Version of symptons questionnaire modified by Dracup K and Moser DK. Beyond sociodemographics: Factors influencing the decision to seek treatment for symptoms of acute myocardial infarction. (DOCX 15 kb)

## Abbreviations

95% CI: 95% confidence intervals
ACS: Acute coronary syndromes
CL: Catheterization laboratory
D2B: Door-to-balloon time
D2D: Door-to-door time
D2Page: CL activation time
DT: Decision time
ECG: Electrocardiogram
EMS: Emergency medical services
ITQ: Interquartile ranges
OR: Odds ratios
PCI: Percutaneous coronary intervention
PDT: Patient delay time
PHT: Pre-hospital time
RT: Reperfusion time
SD: Standard deviation
SDT: System delay time
STEMI: ST-segment elevation acute myocardial infarction
