# Supplementary material for: Symptoms awareness, emergency medical service utilization and hospital transfer delay in myocardial infarction
Source: BMC Health Serv Res. 2018 Jun 25;18:490. doi: 10.1186/s12913-018-3312-6 (PMC6020233; doi:10.1186/s12913-018-3312-6)
Supplement: Supplementary file 1 — Response to Symptoms Questionnaire (English version). Version of symptons questionnaire modified by Dracup K and Moser DK. Beyond sociodemographics: Factors influencing the decision to seek treatment for symptoms of acute myocardial infarction. (DOCX 15 kb) [file 12913_2018_3312_MOESM1_ESM.docx]

Additional file 1

**Manuscript: Symptoms Awareness, Emergency Medical Service Utilization and Hospital Transfer Delay in Myocardial Infarction**

Cézar E. Mesas, Ricardo J. Rodrigues, Arthur E. Mesas, Vivian B. R. Feijó, Lucas M. C. Paraiso, Gabriela F. G. A. Bragatto, Viviane Moron, Marcos H. Bergonso, Laercio Uemura, Cintia M. C. Grion

**Response to Symptoms Questionnaire** – version modified by Dracup K and Moser DK. Beyond sociodemographics: Factors influencing the decision to seek treatment for symptoms of acute myocardial infarction. Heart Lung 1997; 26: 253-62.

**Initial response to acute myocardial infarction symptoms**

( ) Tried to relax

( ) Hoped or prayed symptoms would go away

( ) Pretended nothing was wrong or tried to think of something else

( ) Told a family member

( ) Self-medicated

( ) Tried to alleviate symptoms with position

( ) Told a coworker

( ) Went to a physician's office or hospital

( ) They or family member called physician

( ) They or family member called 911

( ) Told a friend or stranger

( ) Did nothing because feared would die

( ) Other

**Social factors**

Setting where symptoms occurred

( ) Home

( ) Outside home

Witness to symptom onset

( ) Alone

( ) Spouse/family member

( ) Coworker/friend/other

Response ol others to symptom onset*

( ) Suggesting seeking help or called for help

( ) Other behavior

**Cognitive and emotional responses**

Pain severity

( ) 1-7

( ) 8-10

Assessed symptoms as intermittent

( ) Yes

( ) No

Appraisal of symptom seriousness

( ) Not serious

( ) Serious

Symptom attribution

( ) Heart

( ) Others

Had knowledge of MI symptoms

( ) Yes

( ) No

Recognized symptoms as important

( ) Yes

( ) No

Waited for symptoms to go away

( ) Yes

( ) No

Was embarrassed to seek help

( ) Yes

( ) No

Worried about troubling others so didn't ask for help

( ) Yes

( ) No

Feared consequences of seeking help

( ) Yes

( ) No
