# Supplementary material for: Symptoms awareness, emergency medical service utilization and hospital transfer delay in myocardial infarction
Source: BMC Health Serv Res. 2018 Jun 25;18:490. doi: 10.1186/s12913-018-3312-6 (PMC6020233; doi:10.1186/s12913-018-3312-6)
Supplement: Supplementary file 2 — Response to Symptoms Questionnaire (Portuguese version). Version of symptons questionnaire modified by Dracup K and Moser DK. Beyond sociodemographics: Factors influencing the decision to seek treatment for symptoms of acute myocardial infarction. (DOCX 15 kb) [file 12913_2018_3312_MOESM2_ESM.docx]

Additional file 2

**Manuscript: Symptoms Awareness, Emergency Medical Service Utilization and Hospital Transfer Delay in Myocardial Infarction**

Cézar E. Mesas, Ricardo J. Rodrigues, Arthur E. Mesas, Vivian B. R. Feijó, Lucas M. C. Paraiso, Gabriela F. G. A. Bragatto, Viviane Moron, Marcos H. Bergonso, Laercio Uemura, Cintia M. C. Grion

Response to Symptoms Questionnaire –

free translation to Brazilian Portuguese

1. Início dos sintomas:

______/______/______ (_______:_______)

Local:

( ) Domicílio

( ) Local público

( ) Outros

Presenciado:

( ) Sim

( ) Não

Se sim, quem presenciou:

( ) Cônjuge

( ) Outro familiar

( ) Conhecido

( ) Desconhecido

Resposta dos outros aos sintomas (quando aplicável):

( ) Sugeriram buscar ajuda ou buscaram ajuda

( ) Outras atitudes

2. Decisão de buscar ajuda:

______/______/______ (_______:_______)

3. Resposta inicial aos sintomas:

( ) Tentou relaxar

( ) Esperou passar

( ) Não é nada/pensou em outra coisa

( ) Contou a um familiar

( ) Tomou algum remédio

( ) Tentou mudar de posição

( ) Contou a um colega

( ) Contou a um estranho

( ) Procurou hospital ou clínica

( ) Chamou ambulância

( ) Outros chamaram médico/hospital

( ) Outros chamaram ambulância

( ) Outras respostas

4. Respostas cognitivas e emocionais:

( ) “Aconteceu outras vezes e sempre passou”

( ) “Achei que não era sério”

( ) “Achei que não fosse o coração”

( ) “Não sabia que era importante procurar ajuda rápido”

( ) “Não queria incomodar as pessoas”

( ) “Daria muito trabalho ir ao hospital”

( ) “O atendimento no hospital é sempre muito demorado”

( ) “Estava muito fraco, cansado”

( ) “Fiquei com medo”

( ) “Não acho que tenha demorado”

( ) Outros ______________________________________________________________________________

Severidade da dor (de 1 a 10):________
